# Evaluation of IL-29 in Euthyroid Patients with Graves' Orbitopathy: A Preliminary Study

**DOI:** 10.1155/2020/4748612

**Published:** 2020-07-09

**Authors:** Bogusz Falkowski, Ewelina Szczepanek-Parulska, Nadia Sawicka-Gutaj, Aleksandra Krygier, Marek Ruchala

**Affiliations:** Department of Endocrinology, Metabolism and Internal Medicine, Poznan University of Medical Sciences, Przybyszewskiego 49, 60-355 Poznan, Poland

## Abstract

**Background:**

The most frequent cause of hyperthyroidism is Graves' disease (GD). Orbitopathy is the most prevalent and recognizable extrathyroidal manifestation of Graves' disease with unrevealed pathogenesis. Interleukin 29 (IL-29) is a relatively newly discovered inflammatory cytokine. Thus, the aim of this study was to evaluate the relationship between IL-29 and Graves' orbitopathy (GO) in euthyroid patients.

**Methods:**

Thirty-one euthyroid patients with Graves' disease and with active GO [clinical activity score (CAS) ≥ 3/7], seventeen euthyroid patients with GD but without GO, and seventy-two healthy control subjects (CS) matched for age and gender were enrolled in the study. The following parameters were evaluated in every participant: thyroid-related hormones and autoantibodies and inflammatory markers (white blood cells, hsCRP). ELISA assay was applied to measure the concentration of IL-29.

**Results:**

We found higher level of IL-29 in GO group in comparison with CS [165 (133-747) vs. 62 (62-217) pg/mL, *p* < 0.001]. Furthermore, participants in the subgroup with GD with GO as compared with GD without GO had higher concentration of IL-29 [165 (133-747) vs. 62 (62-558) pg/mL, *p* = 0.031]. The ROC analysis for IL-29 revealed IL-29 cut-off of 105 pg/mL (sensitivity 1.000 and specificity 0.597) as the best value significantly indicating the presence of GO in GD [area under the ROC curve (AUC): 0.739, 95% confidence interval (CI): 0.646-0.833, *p* < 0.001].

**Conclusions:**

The present study revealed for the first time an elevated level of IL-29 in the serum of patients with GD and GO that might suggest its involvement in the pathogenesis of GD ocular complications.

## 1. Introduction

Graves' disease (GD) is recognized as the most common cause of hyperthyroidism, and the risk of development of GD during the whole life is estimated at 3% in women and 0.5% in men [1], and anti-TSH receptor antibodies (TRAb) play the main role in the pathogenesis of GD. Graves' orbitopathy (GO), also called ophthalmopathy, is an extrathyroidal manifestation of GD affecting the eye muscles and retroorbital fat. In European population, the prevalence of GO is estimated about 1 case per 1000 people [2]. Activity of GO can be assessed using clinical activity score (CAS), and CAS ≥ 3/7 indicates an active GO. Severity can be expressed in simple staging system as mild, moderate to severe, and sight threatening (sometimes called very severe) [3]. It is estimated that about 5% of patients with GD suffer from moderate-to-severe GO [4]. The main treatment option for GO is therapy with corticosteroids. In the last few years, some new methods were applied to treat GO: rituximab (a monoclonal antibody against the protein CD20 on the surface of B cells) [5], tocilizumab [interleukin-6 (IL-6) receptor antibodies] [6], and teprotumumab (monoclonal antibody being an inhibitor of insulin-like growth factor I receptor) [7]. They resulted from recently discovered new immune pathways which provided a basis to develop new treatment options. Unfortunately, effectiveness of mentioned drugs is limited, so new medications are still searched for.

Interleukin 29 (IL-29) is also recalled as interferon lambda 1 (IFN-*λ*1). This protein is quite a new member of the recently discovered IFN-*λ* family and plays a strong antiviral role [8]. Moreover, it is known that, without any exposure to viruses, dendritic cells and macrophages produce Il-29 during wide range of diseases with autoimmune aetiology [9]. The elevated levels of IL-29 were already detected in some autoimmune diseases such as Sjögren syndrome, rheumatoid arthritis, systemic sclerosis, systemic lupus erythematosus, and psoriasis [10–14]. Moreover, elevated concentrations of IL-29 were found in atopic dermatitis and asthma [15, 16]. Other interleukins, such as IL-10, IL-19, IL-20, IL-22, IL-24, IL-26, IL-28A, and IL-28B, are closely related to IL-29. They together form a large family called IL-10 family [17]. We know now that polymorphisms of the genes of IL-10, IL-22, and IL-28 are associated with a higher prevalence of autoimmune thyroid disease [18–20]. Until the present study was conducted, only one research aimed to evaluate the role of IL-29 in thyroid disorders. In the cited study, elevated serum levels of IL-28 and IL-29 were detected in patients with Hashimoto's thyroiditis (HT) [20].

The concentration of IL-29 in Graves' orbitopathy has not been evaluated yet. The most valuable results can be drawn from the observation of IL-29 in patients with Graves' orbitopathy in euthyroidism. Thus, the aim of the present study was to assess the concentration of IL-29 in euthyroid patients with Graves' orbitopathy in comparison with the healthy controls and euthyroid patients with Graves' disease without orbitopathy.

## 2. Patients and Methods

### 2.1. Patients' Selection

**Figure 1:**
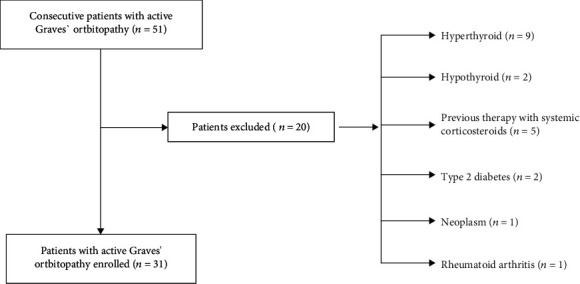
Study flow chart.

This study was designed as a single-center cross-sectional with consecutive enrollment. The final study sample included 31 euthyroid patients with GD and active GO [aged 53 (46-59) years], of which 22 (71%) were women and 17 euthyroid patients with GD without GO [aged 46 (35-51) years], of which 12 (71%) were women, referred to the tertiary reference endocrine center. Every participant was European Caucasian, aged between 18 and 75 years, without liver failure [alanine transaminase (AST) and/or aspartate transaminase (ALT) 1.5 times the upper norm] and had estimated with The Chronic Kidney Disease Epidemiology Collaboration (CKD-EPI) equation glomerular filtration rate (eGFR) >60 mL min^−1^1.73 m^−2^. Detailed inclusion and exclusion criteria are presented in Figure 1. Control group (CS) comprised 72 healthy volunteers [aged 46 (31-61) years], matched for age and gender, of which 52 (72%) were women. CS were examined clinically (no sign of thyroid or other inflammatory disorders), using ultrasound (US) imaging (normal result), and underwent the same laboratory tests as the study group (all results within reference interval).

All experimental protocols are in accordance with the 1975 Declaration of Helsinki and were approved by the Bioethical Committee of Poznan University of Medical Sciences (approval numbers: 174/17 and 87/19). Every participant was involved voluntarily and delivered a written informed consent to participate in the study. All data needed to reproduce the results of this study are available from the corresponding author upon reasonable request.

### 2.2. Graves' Disease and Graves' Orbitopathy Assessment

Graves' disease was diagnosed if the following criteria were fulfilled: clinical symptoms of hyperthyroidism and/or characteristic US image (increased size of thyroid gland, echotexture hypoechoic or heterogeneous, and hypervascular on color Doppler) and biochemical indicators (low thyrotropin-TSH, high free triiodothyronine-FT3, high free thyroxine-FT4, and elevated anti-TSH receptor antibodies-TRAb) [21]. Mentioned information was taken from the hospital database or patients' anamnesis. Moreover, every patient underwent an ophthalmological examination of GO in accordance with the European Group on Graves' orbitopathy (EUGOGO) guidelines [22, 23]. The actual severity of GO was assessed using clinical activity score (CAS), where CAS ≥ 3/7 indicated an active GO. Moreover, to confirm the diagnosis of GO, magnetic resonance imaging (MRI) of orbits was performed. Patients in our study were already treated with antithyroid medications and euthyroid, which means that TSH, FT3, and FT4 were within the normal range. Every patient with GO enrolled in our research had GD and active GO.

### 2.3. Laboratory Analysis

Blood samples were obtained after overnight fasting and before the ingestion of L-thyroxine (L-T4) in case it was supplemented. Every patient had the following laboratory tests performed on serum: alanine transaminase (ALT), aspartate transaminase (AST), creatinine, and high-sensitivity C-reactive protein (hsCRP) using enzymatic assays on a Hitachi Cobas e501 (Roche Diagnostics, Indianapolis, IN, USA) analyzer. The estimated glomerular filtration rate (eGFR) was calculated afterwards using The Chronic Kidney Disease Epidemiology Collaboration (CKD-EPI) formula from creatinine serum level, age, and sex [24]. White blood cells (WBC) were measured with Sysmex-XN 1000 (Sysmex Europe GmbH, Bornbarch, Germany) analyzer. Electrochemiluminescence was used to measure serum concentrations of TSH, FT4, FT3, antithyroid peroxidase antibodies (TPOAb), and antithyroglobulin antibodies (TGAb) on Hitachi Cobas e601 analyzer (Roche Diagnostics, Indianapolis, IN, USA). TRAb was assessed using radioimmunoassay [TRAK RIA kits (Brahms GmbH, Hennigsdorf, Germany)]. Applied tests have the following normal ranges of TSH 0.27-4.2 *μ*IU/mL, FT4 11.5-21 pmol/L and FT3 3.9-6.7 pmol/L, TPOAb <34 IU/mL, TGAb 10-115 IU/mL, and TRAb <2 IU/L. The level of IL-29 was measured using IL-29 ELISA Kit (intra-assay CV <5.5%, interassay CV <6.5%; Aviva Systems Biology, San Diego, USA) [25]. Laboratory analyses were performed on the same day when blood was taken. Only concentrations of IL-29 were measured from the serum, previously stored frozen at −80°C, after all samples were collected.

### 2.4. Statistical Analysis

The acquired data were analysed and presented statistically using Statistica V13 (StatSoft, Tulsa, Oklahoma, USA, RRID: SCR_014213, https://www.tibco.com). At first, normality was analyzed by Shapiro-Wilk test. The majority of qualitative variables did not follow normal distribution (only distributions of FT3 and FT4 were normal), which determined the use of nonparametric statistical tests. Qualitative parameters are expressed as median with 25-75% interquartile range (IQR). In descriptive characteristics, two groups were compared using nonparametric Mann–Whitney *U* test (except sex which was compared using Chi-squared test). The Spearman's rank correlation coefficients were calculated to estimate the correlations. Additionally, IL-29 was also evaluated in receiver operating characteristic (ROC) analysis with the Youden index to determine the cut-off [26]. The *p* value threshold was set as statistically significant in every analysis at <0.05.

## 3. Results

Studied group comprised 31 participants diagnosed with GD with GO, 17 participants with GD without GO, and the control group included 72 healthy subjects (CS). A comparison of basic parameters is provided in Table 1. CS were matched for age and gender. Compared groups did not differ according to markers of inflammation, such as CRP and WBC.

**Figure 2:**
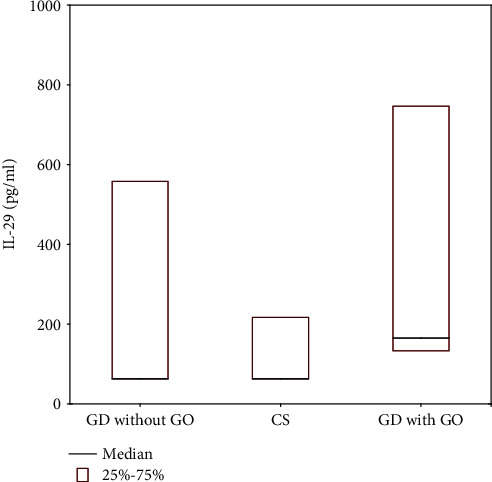
IL-29 in Graves' disease without orbitopathy—GD without GO, Graves' disease with GO—GD with GO and control subjects—CS. Values are expressed as median and interquartile range (IQR).

Figure 2 shows median concentration of IL-29 in Graves' disease without orbitopathy—GD without GO, Graves' disease with GO—GD with GO, and control subjects—CS. Values are expressed as median and 25-75% interquartile range (IQR). Participants in the subgroup with GO as compared with CS had higher IL-29 concentration [165 (133-747) vs. 62 (62-217) pg/mL, *p* < 0.001]. Participants in the subgroup with GO as compared with GD without GO had higher IL-29 concentration [165 (133-747) vs. 62 (62-558) pg/mL, *p* = 0.031] (Table 2).

Statistically significant positive correlation was found between IL-29 level and WBC (*r* = 0.45, *p* = 0.012) in Graves' disease with GO. Age, ALT, AST, and hsCRP did not correlate statistically significantly with concentration of IL-29 neither in patients with GD and GO nor with GD without GO.

**Figure 3:**
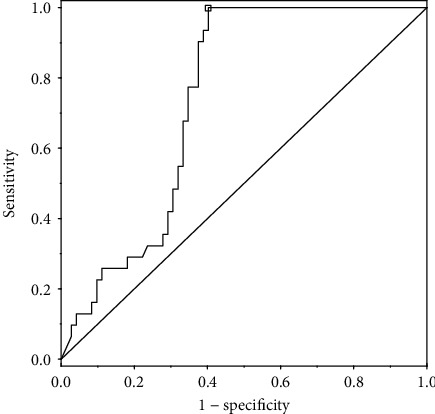
ROC curve for the prediction of GO occurrence using IL-29.

Figure 3 shows ROC curve for the prediction of GO occurrence using IL-29. The ROC analysis for IL-29 at GO diagnosis revealed IL-29 cut-off of 105 pg/mL (Youden index 0.60; sensitivity 1.000 and specificity 0.597) as the best value significantly indicating the presence of GO [area under the ROC curve (AUC): 0.739, 95% confidence interval (CI): 0.646-0.833, *p* < 0.001].

## 4. Discussion

In the present study, we demonstrated that increased serum level of IL-29 was associated with active GO in euthyroid patients with GD. Moreover, increased serum concentration of IL-29 was found in euthyroid patients with GD and GO as compared with euthyroid patients with GD and simultaneously without GO. To the best of our knowledge, our study is the first evaluating IL-29 serum concentrations in GO. We assessed patients in the euthyroid state, which excludes the potential influence of thyroid hormones on the level of IL-29. What is more, very high sensitivity in the ROC analysis for IL-29 at GO diagnosis suggests potential of Il-29 in diagnostics. In the future, IL-29 can be a potential point of action of the novel drugs for GO. Taken together, our results might suggest the potential relationship between the thyroid autoimmunity and IL-29, which can be very useful in clinical setting.

In the discussion of the results of the present study, the most important is single report focused on IL-29 in HT conducted on 99 patients [20]. The cited study revealed increased IL-29 serum level in HT patients if compared with healthy controls. It suggests the involvement of IL-29 in the pathogenesis of autoimmune thyroid disease. As mentioned above, there are no other studies which assessed the concentration of IL-29 in Graves' disease; thus, we are unable to compare present results with any more studies.

Pathogenesis of GO is still not fully determined, which results in limited therapeutic modalities [27]. We know some inflammatory mediators which gene overexpression was found in orbits of patients with GO. In one study, eye muscles and retroorbital fat tissue were examined as they are two major sites involved in the development of GO. The severity of the inflammatory process positively correlated with TNF-*α* mRNA and IL-6 mRNA expression and negatively with IL-4 mRNA and IL-10 mRNA expression [28]. Furthermore, some pathways were already used in clinical trials: tocilizumab [interleukin-6 (IL-6) receptor antibodies] [6] and teprotumumab (monoclonal antibody being an inhibitor of insulin-like growth factor I receptor) [7]. The results of mentioned trials are very promising. What is more, also, IL-7, IL-8, IL-15, IL-16, and IL-17 are engaged in GO pathogenesis that suggests multifactorial background [29–32].

The action of IL-29 on eye muscle and retroorbital fat tissue can occur via some signaling pathways. Potential pathways that take part in GO development include nuclear factor-kappa B (NF-*κ*B) signaling pathway [33], which is induced by IL-29 [34]. IL-29 is also an activator of Janus kinase/signal transduction and activator (JAK-STAT) through STAT1, STAT2, STAT3, and STAT5 [35]. What is essential, thyroid-associated orbitopathy is an effect of the activation of JAK-STAT [36]. Moreover, IL-29 is an inductor of mitogen-activated protein kinase (MAPK), and MAPK is possibly engaged in GD development [37, 38]. IL-29 also affects B lymphocytes, which are responsible for TRAb production [39]. This might explain the correlation of TRAb and IL-29 observed in our study. Another action of IL-29 is to stimulate monocytes to excretion of IL-6, IL-8, IL-10, and IL-8 which is proved to be strongly associated with GD [40, 41].

Some limitations of the present study should be mentioned. This is a preliminary study on relatively small sample size. Relatively small number of participants is a result of restrictive inclusion criteria such as full biochemical euthyroidism in every enrolled patient. Moreover, patients in the group with GD and GO were older than patients in group with GD and without GO, but age did not correlate statistically significantly with concentration of IL-29 that excludes potential bias. Furthermore, any judgments about causality between IL-29 and GO cannot be made as we had a single point of measurements. However, the strengths of the present study must be addressed too. There are strict inclusion and exclusion criteria that eliminate wide range of possible biases. Enrollment of only euthyroid subjects allowed us to examine the impact of autoimmunity without bias in the form of hormonal imbalance.

What is also essential, applied methods are minimally invasive and widely available that makes our results replicable and easy to introduce in clinical practice in the future. The present study can serve as a great background to plan a prospective study evaluating the relationship between IL-29 and GO.

## 5. Conclusions

The present study revealed for the first time an elevated level of IL-29 in the serum of patients with GD and GO that might suggest its involvement in the pathogenesis of GD ocular complications. The potential role of IL-29 as a predictor of GO occurrence needs to be confirmed in studies conducted on larger cohorts of GO patients. Moreover, future investigations are required to evaluate the clinical potential of IL-29 as a therapeutic target in GO.

## Figures and Tables

**Table 1:** Clinical and biochemical characteristics of the study participants with Graves' disease and Graves' orbitopathy—GD with GO vs. control subjects—CS.

Parameter	Values		*p* value
	GD with GO (*n* = 31)	CS (*n* = 72)	
Age (years)	53 (46-59)	46 (31-61)	0.067
Male sex, *n* (%)	9 (29)	20 (28)	0.897^1^
WBC, 1000/*μ*L	6.59 (5.89-7.52)	6.37 (5.25-7.83)	0.217
hsCRP (mg/L)	1.5 (0-3.8)	1.1 (0.4-2.5)	0.613
ALT (U/L)	20 (13-26)	20 (13-27)	0.863
AST (U/L)	19 (16-26)	19 (15-23)	0.580
TPOAb (U/mL)	174 (58-259)	12 (9-16)	*<0.001*
TGAb (U/mL)	133 (27-267)	11 (10-16)	*<0.001*
TRAb (U/L)	13.56 (4.29-27.8)	0.30 (0.19-0.49)	*<0.001*
TSH (*μ*IU/mL)	1.38 (0.65-2.08)	1.63 (1.11-2.17)	0.178
FT3 (pmol/L)	4.3 (3.9-5.23)	4.93 (4.51-5.50)	*<0.001*
FT4 (pmol/L)	17.8 (13.6-19.31)	16.13 (14.82-17.61)	0.159
IL-29 (pg/mL)	165 (133-747)	62 (62-217)	*<0.001*

Data are presented in the form of number (%) or median (IQR).

The italic *p* values are those which are statistically significant.

^1^Chi-squared test; Mann–Whitney *U* test in other cases, where it is not marked with the superscript one.

ALT: alanine transaminase; AST: aspartate transaminase; FT3: free triiodothyronine; FT4: free thyroxine; hsCRP: high-sensitivity C-reactive protein; IQR: interquartile range; TGAb: antithyroglobulin antibodies; TPOAb: antithyroid peroxidase antibodies; TRAb: anti-TSH receptor antibodies; TSH: thyroid-stimulating hormone; U: unit; WBC: white blood cells.

**Table 2:** Clinical and biochemical characteristics of the study participants with Grave's disease with Graves' orbitopathy—GD with GO vs. participants with Graves' disease without GO—GD without GO.

Parameter	Values		*p* value
	GD with GO (*n* = 31)	GD without GO (*n* = 17)	
Age (years)	53 (46-59)	46 (35-51)	*0.006*
Male sex, *n* (%)	9 (29)	5 (29)	0.761^1^
WBC, 1000/*μ*L	6.59 (5.89-7.52)	7.34 (6.22-8.37)	0.253
hsCRP (mg/L)	1.5 (0-3.8)	1.0 (0.4-1.6)	0.281
ALT (U/L)	20 (13-26)	17 (15-25)	0.796
AST (U/L)	19 (16-26)	18 (16-22)	0.813
TPOAb (U/mL)	174 (58-259)	56 (27-114)	*0.033*
TGAb (U/mL)	133 (27-267)	32 (10-251)	0.203
TRAb (U/L)	13.56 (4.29-27.8)	1.79 (0.89-2.92)	*<0.001*
TSH (*μ*IU/mL)	1.38 (0.65-2.08)	1.45 (0.68-2.06)	0.872
FT3 (pmol/L)	4.3 (3.9-5.23)	4.67 (4.46-5.2)	*0.025*
FT4 (pmol/L)	17.8 (13.6-19.31)	15.81 (14.43-16.02)	0.126
IL-29 (pg/mL)	165 (133-747)	62 (62-558)	*0.031*

Data are presented in the form of number (%) or median (IQR).

The italic *p* values are those which are statistically significant.

^1^Chi-squared test with Yates's correction; Mann–Whitney *U* test in other cases, where it is not marked with the superscript one.

ALT: alanine transaminase; AST: aspartate transaminase; FT3: free triiodothyronine; FT4: free thyroxine; hsCRP: high-sensitivity C-reactive protein; IQR: interquartile range; TGAb: antithyroglobulin antibodies; TPOAb: antithyroid peroxidase antibodies; TRAb: anti-TSH receptor antibodies; TSH: thyroid-stimulating hormone; U: unit; WBC: white blood cells.

## Data Availability

The data used to support the findings of this study may be released upon application to the corresponding author, who can be contacted at the address Department of Endocrinology, Metabolism and Internal Medicine, Przybyszewskiego 49, 60-355, Poznan, Poland, and at the e-mail: bogusz.falkowski@onet.pl.
